# Supplementary material for: Growth-dependent Gene Expression Variation Influences the Strength of Codon Usage Biases
Source: Mol Biol Evol. 2023 Aug 24;40(9):msad189. doi: 10.1093/molbev/msad189 (PMC10482319; doi:10.1093/molbev/msad189)
Supplement: msad189_Supplementary_Data [file msad189_supplementary_data.pdf]

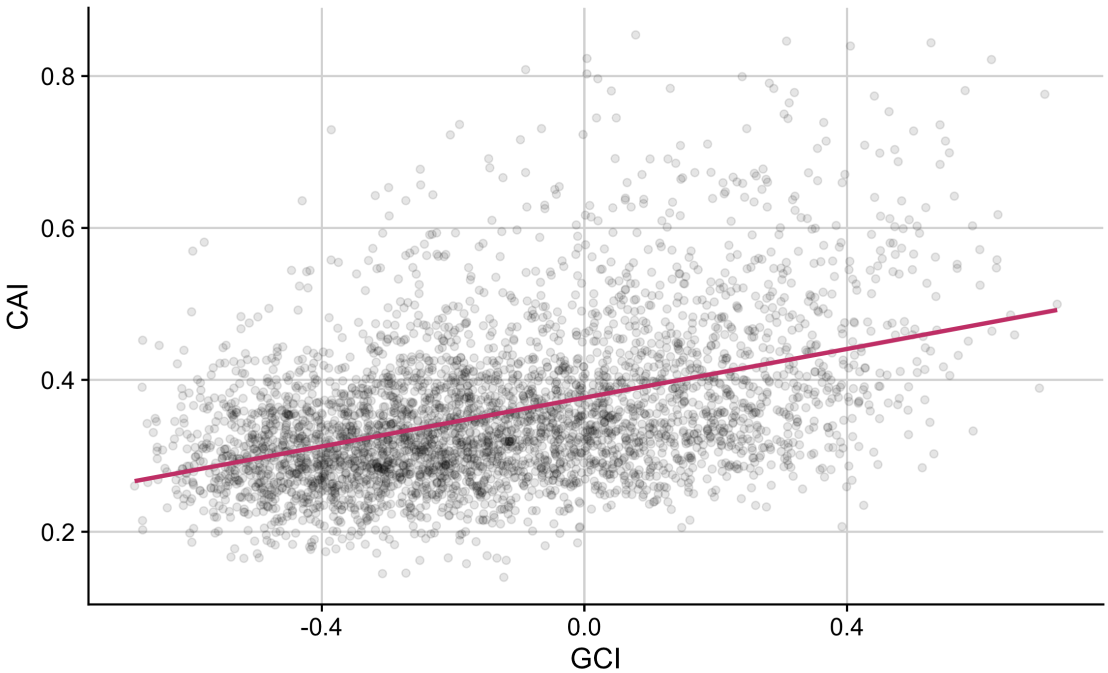

Supplementary Figure S1: **Growth Correlation Index (GCI) has a significant linear relationship with Codon Adaptation Index (CAI).** For each gene in the full *E. coli* data set, CAI values are shown against GCI values. The performance of this model relative to models incorporating mean expression data is shown in Figure 5 in the main text.

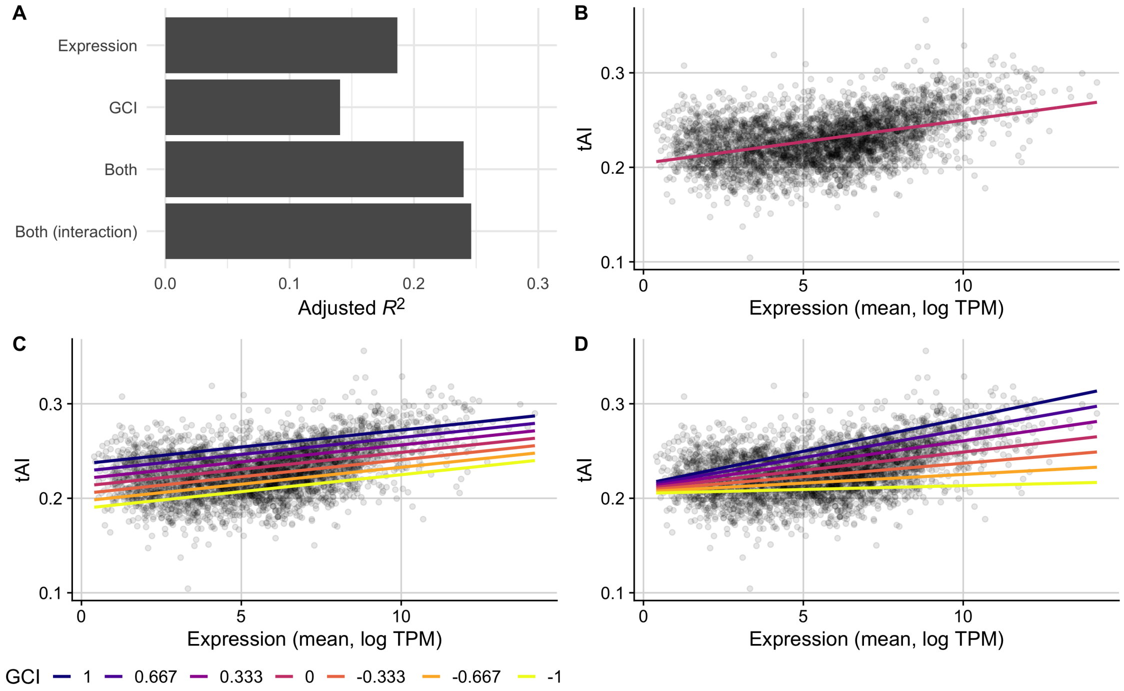

Supplementary Figure S2: **tRNA Adaptation Index (tAI) is partially predicted by Growth Correlation Index (GCI) in the full *E. coli* RNA data set.** A comparison of the predictive ability (measured as R^2 after adjustment) of linear models that use either: 1) mean expression values, 2) GCI values, 3) both expression and GCI values, or 4) both values with an interaction term, to predict tAI. (B, C, D) tAI against mean expression for the top 3 performing models with observed values for each gene shown as points and model predictions as lines. The fit of model 1, which predicts tAI using only mean gene expression values, is shown with one line (B), while models 3 and 4 are shown with several lines colored by potential fixed GCI values (C and D, respectively).

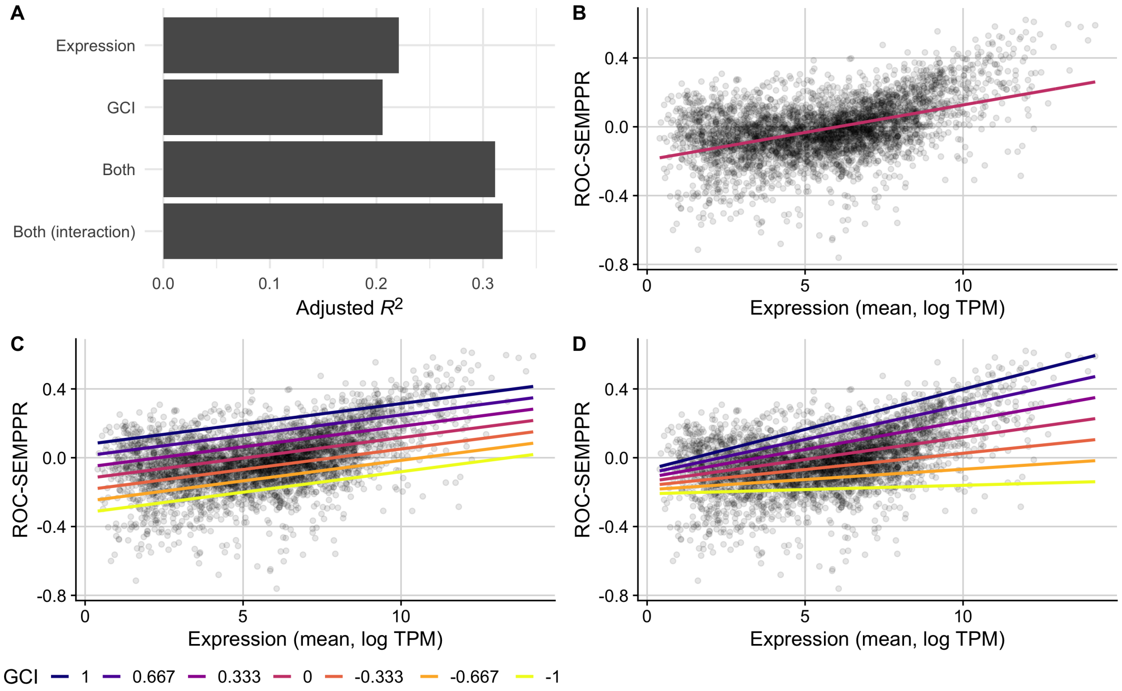

Supplementary Figure S3: **The proportional difference in ribosome overhead costs in a Stochastic Evolutionary Model of Protein Production Rate (ROC SEMPPR) is partially predicted by Growth Correlation Index (GCI) in the full *E. coli* RNA data set.** A comparison of the predictive ability (measured as R^2 after adjustment) of linear models that use either: 1) mean expression values, 2) GCI values, 3) both expression and GCI values, or 4) both values with an interaction term, to predict ROC SEMPPR. (B, C, D) ROC SEMPPR against mean expression for the top 3 performing models with observed values for each gene shown as points and model predictions as lines. The fit of model 1, which predicts ROC SEMPPR using only mean gene expression values, is shown with one line (B), while models 3 and 4 are shown with several lines colored by potential fixed GCI values (C and D, respectively).

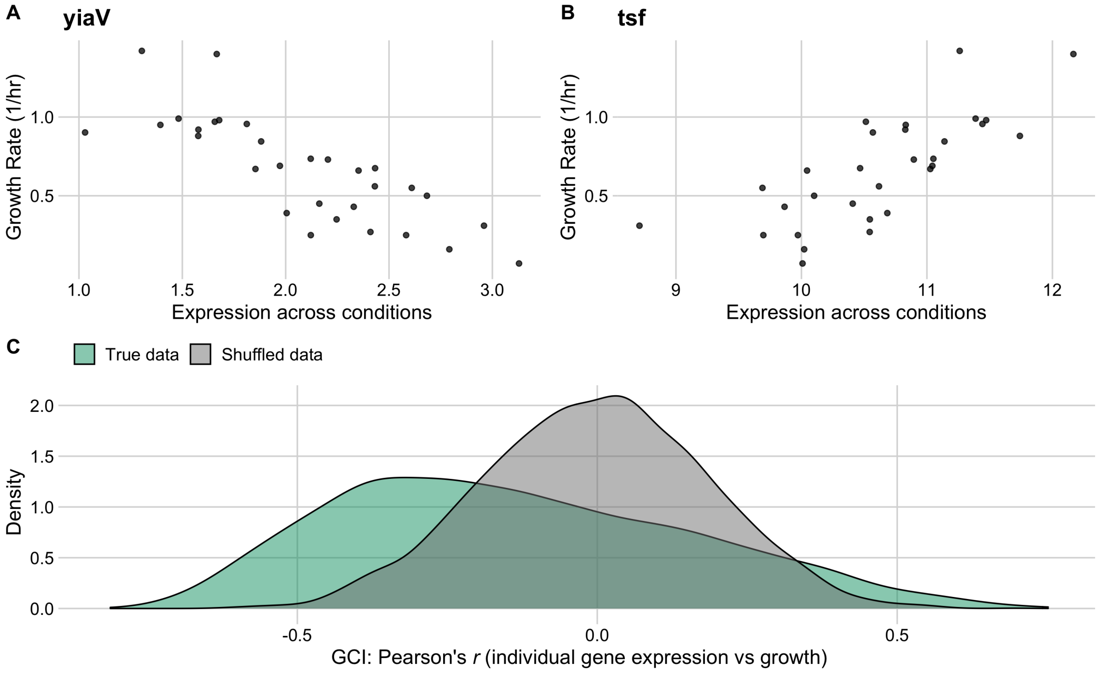

Supplementary Figure S4: **Individual gene expression across conditions variably correlates with growth rate in the sparse *E. coli* RNA data set.** The Growth Correlation Index (GCI) measures the correlation between growth rate and expression for individual genes. The top row shows the two genes with the most negative (yiaV, A) and most positive (tsf, B) correlation between growth rate and expression across all conditions. (C) The distribution of GCI values for all genes (shown in green) against a reference data set with permuted expression and growth data (grey).

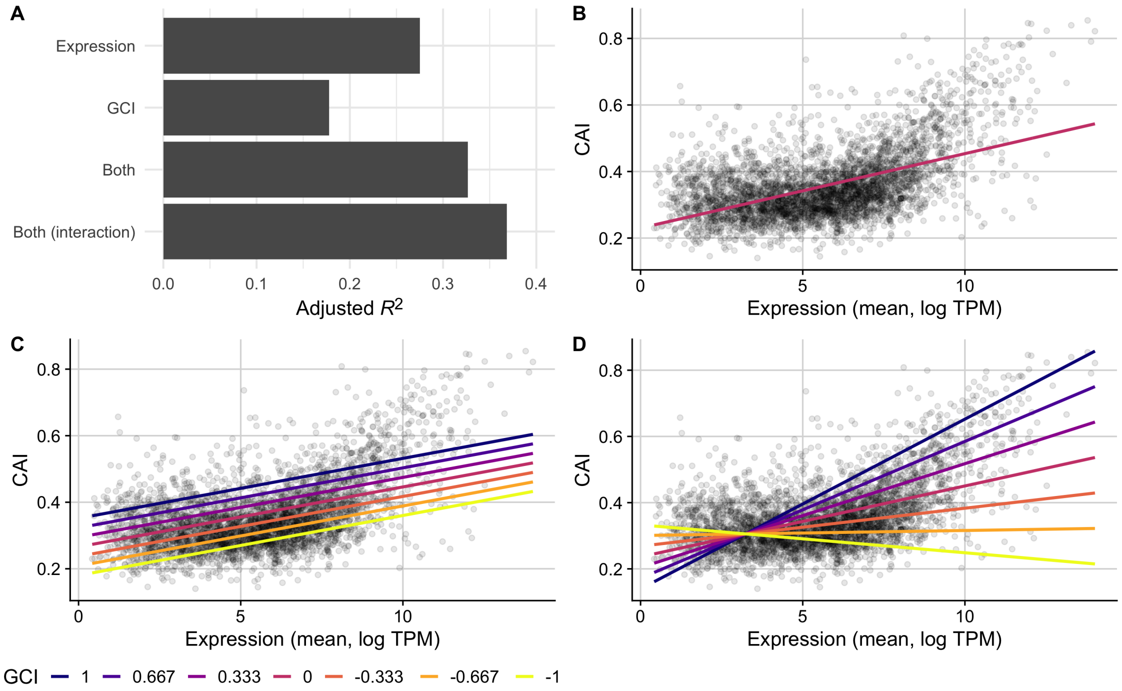

Supplementary Figure S5: **Codon Adaptation Index (CAI) is partially predicted by Growth Correlation Index (GCI) in the sparse *E. coli* RNA data set.** (A) A comparison of the predictive ability (measured as R^2 after adjustment) of linear models that use either: 1) mean expression values, 2) GCI values, 3) both expression and GCI values, or 4) both values with an interaction term, to predict CAI. (B, C, D) CAI against mean expression for the top 3 performing models with observed values for each gene shown as points and model predictions as lines. The fit of model 1, which predicts CAI using only mean gene expression values, is shown with one line (B), while models 3 and 4 are shown with several lines colored by potential fixed GCI values (C and D, respectively). VIF (variance inflation factor) between mean expression and GCI is calculated to be 1.20.

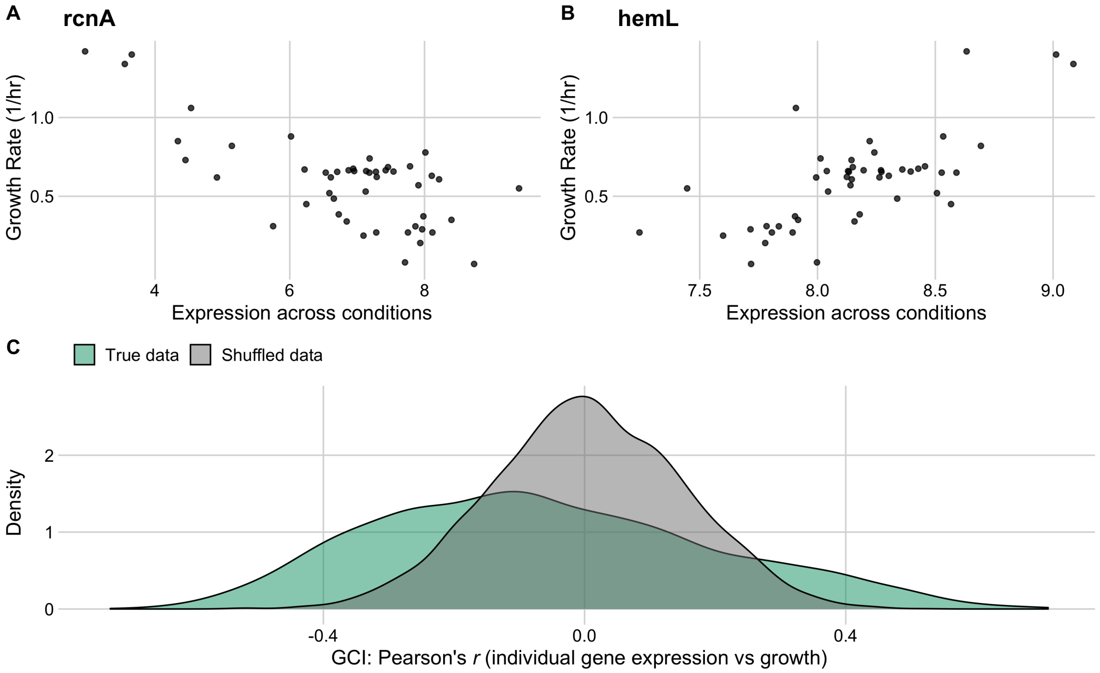

Supplementary Figure S6: **Individual gene expression across conditions variably correlates with growth rate in the neutral *E. coli* RNA data set that excludes adaptive laboratory environment (ALE) strains.** The Growth Correlation Index (GCI) measures the correlation between growth rate and expression for individual genes. The top row shows the two genes with the most negative (rcnA, A) and most positive (hemL, B) correlation between growth rate and expression across all conditions. (C) The distribution of GCI values for all genes (shown in green) against a reference data set with permuted expression and growth data (grey).

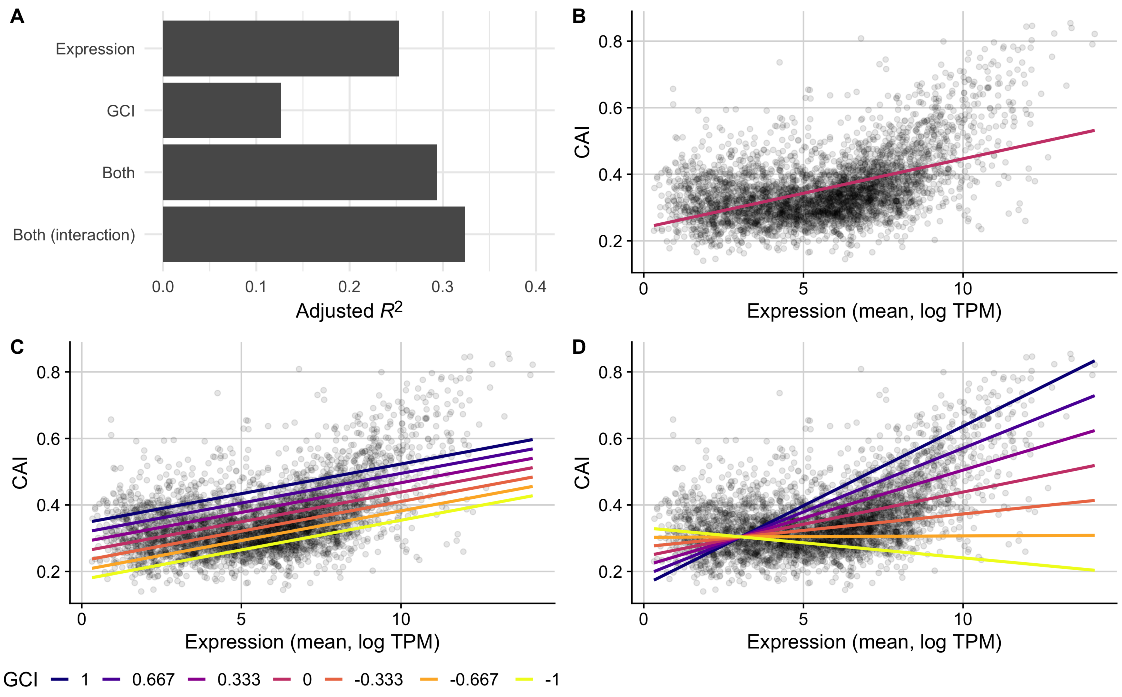

Supplementary Figure S7: **CAI is partially predicted by GCI in the neutral *E. coli* RNA data set that excludes adaptive laboratory environment (ALE) strains.** (A) A comparison of the predictive ability (measured as R^2 after adjustment) of linear models that use either: 1) mean expression values, 2) GCI values, 3) both expression and GCI values, or 4) both values with an interaction term, to predict CAI. (B, C, D) CAI against mean expression for the top 3 performing models with observed values for each gene shown as points and model predictions as lines. The fit of model 1, which predicts CAI using only mean gene expression values, is shown with one line (B), while models 3 and 4 are shown with several lines colored by potential fixed GCI values (C and D, respectively). VIF (variance inflation factor) between mean expression and GCI is calculated to be 1.19.

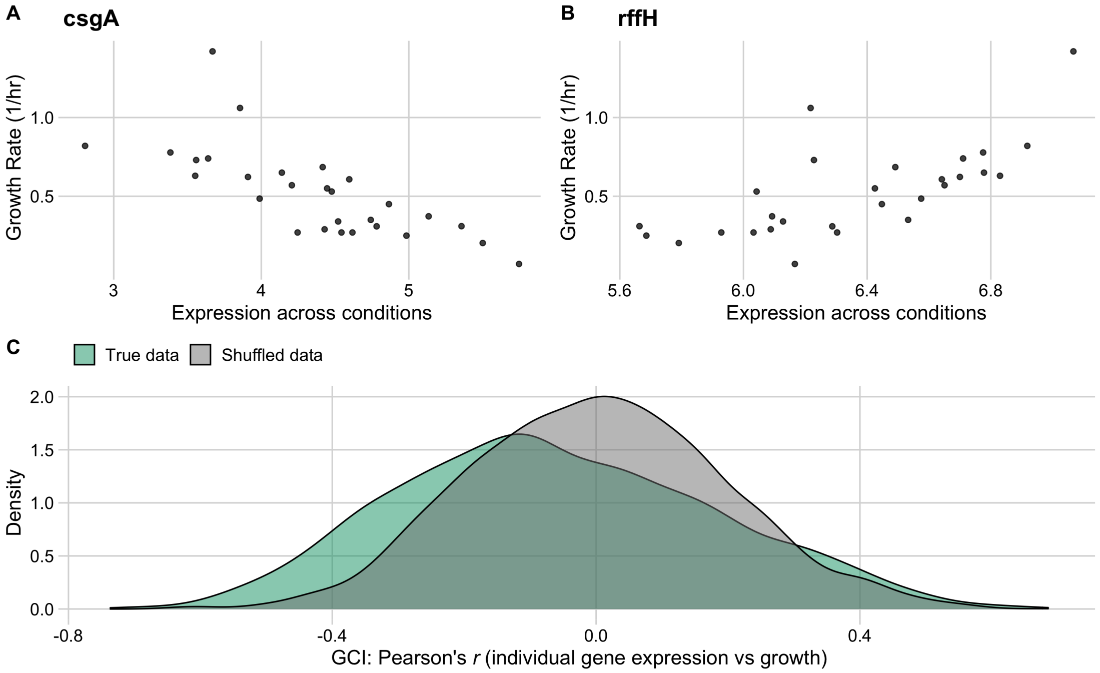

Supplementary Figure S8: **Individual gene expression across conditions variably correlates with growth rate in the neutral *E. coli* RNA data set that excludes ALEs (adaptive laboratory environment strains), mutants, and knock-outs.** The Growth Correlation Index (GCI) measures the correlation between growth rate and expression for individual genes. The top row shows the two genes with the most negative (csgA, A) and most positive (rffH, B) correlation between growth rate and expression across all conditions. (C) The distribution of GCI values for all genes (shown in green) against a reference data set with permuted expression and growth data (grey).

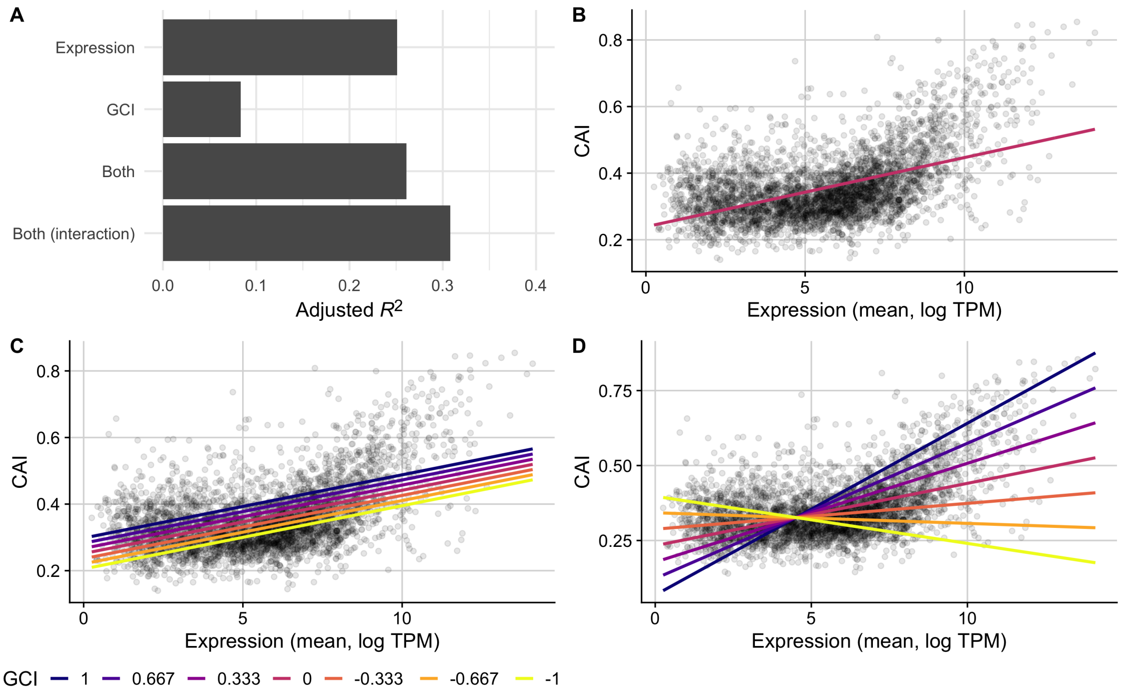

Supplementary Figure S9: **CAI is partially predicted by GCI in the neutral *E. coli* RNA data set that excludes ALEs (adaptive laboratory environment strains), knock-outs, and mutants.** (A) A comparison of the predictive ability (measured as R^2 after adjustment) of linear models that use either: 1) mean expression values, 2) GCI values, 3) both expression and GCI values, or 4) both values with an interaction term, to predict CAI. (B, C, D) CAI against mean expression for the top 3 performing models with observed values for each gene shown as points and model predictions as lines. The fit of model 1, which predicts CAI using only mean gene expression values, is shown with one line (B), while models 3 and 4 are shown with several lines colored by potential fixed GCI values (C and D, respectively). VIF (variance inflation factor) between mean expression and GCI is calculated to be 1.18.

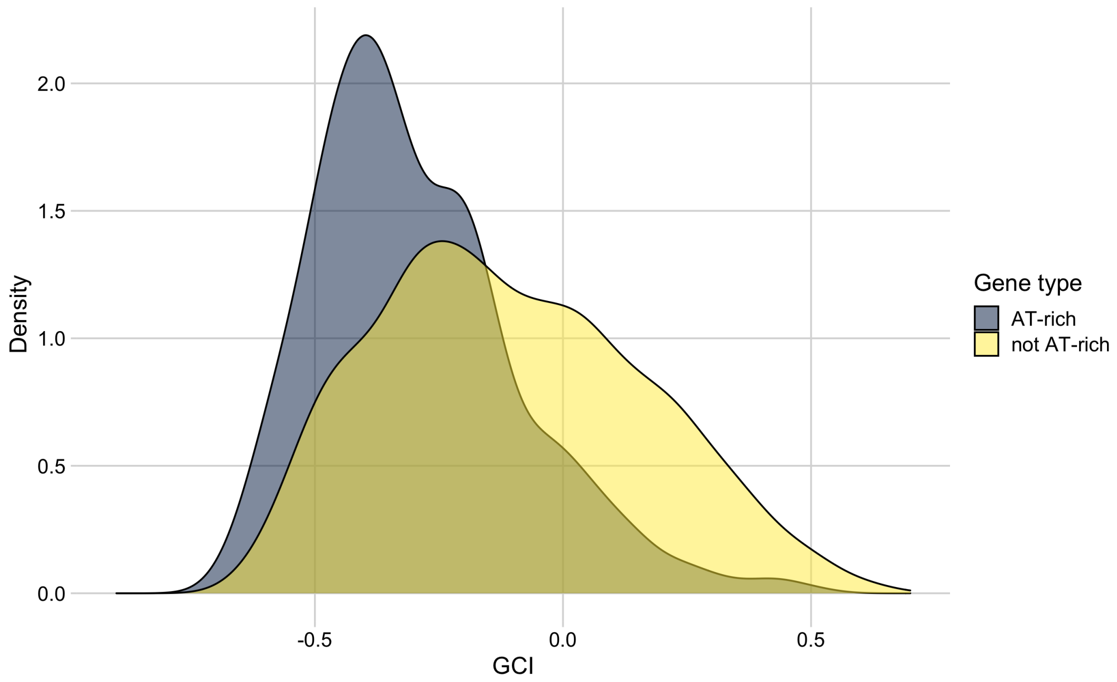

Supplementary Figure S10: **AT-rich genes are skewed towards negative Growth Correlation Index (GCI) values in the full *E. coli* RNA data set.** The distribution of GCI values for group 3, AT-rich genes from dos Reis et al. (2003) (shown in blue) and for all other genes (yellow). The mean GCI values for the two distributions are -0.30 and -0.11, respectively, and they are significantly different (t-test, $p < 10^{-10}$).

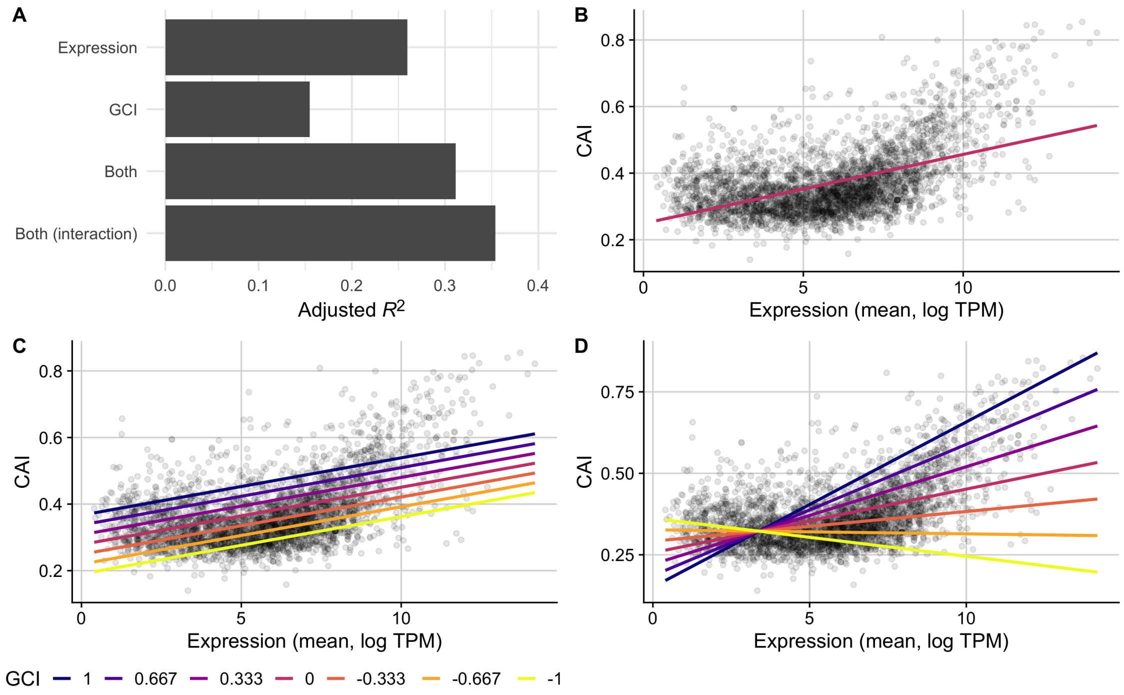

Supplementary Figure S11: **Codon Adaptation Index (CAI) is partially predicted by Growth Correlation Index (GCI) in the *E. coli* RNA data set that excludes AT-rich genes from dos Reis et al. (2003).** (A) A comparison of the predictive ability (measured as R^2 after adjustment) of linear models that use either: 1) mean expression values, 2) GCI values, 3) both expression and GCI values, or 4) both values with an interaction term, to predict CAI. (B, C, D) CAI against mean expression for the top 3 performing models with observed values for each gene shown as points and model predictions as lines. The fit of model 1, which predicts CAI using only mean gene expression values, is shown with one line (B), while models 3 and 4 are shown with several lines colored by potential fixed GCI values (C and D, respectively). VIF (variance inflation factor) between mean expression and GCI is calculated to be 1.15.

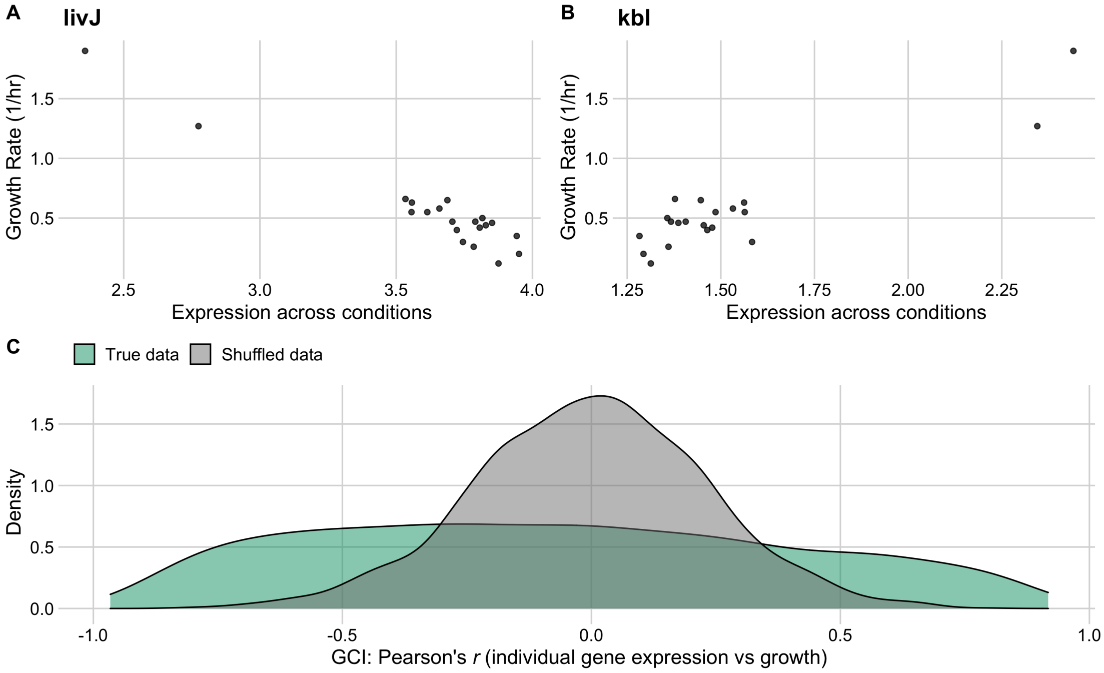

Supplementary Figure S12: **Individual gene expression across conditions variably correlates with growth rate in the *E. coli* protein data set.** The Growth Correlation Index (GCI) measures the correlation between growth rate and expression for individual genes. The top row shows the two genes with the most negative (livJ, A) and most positive (kbl, B) correlation between growth rate and expression across all conditions. (C) The distribution of GCI values for all genes (shown in green) against a reference data set with permuted expression and growth data (grey).

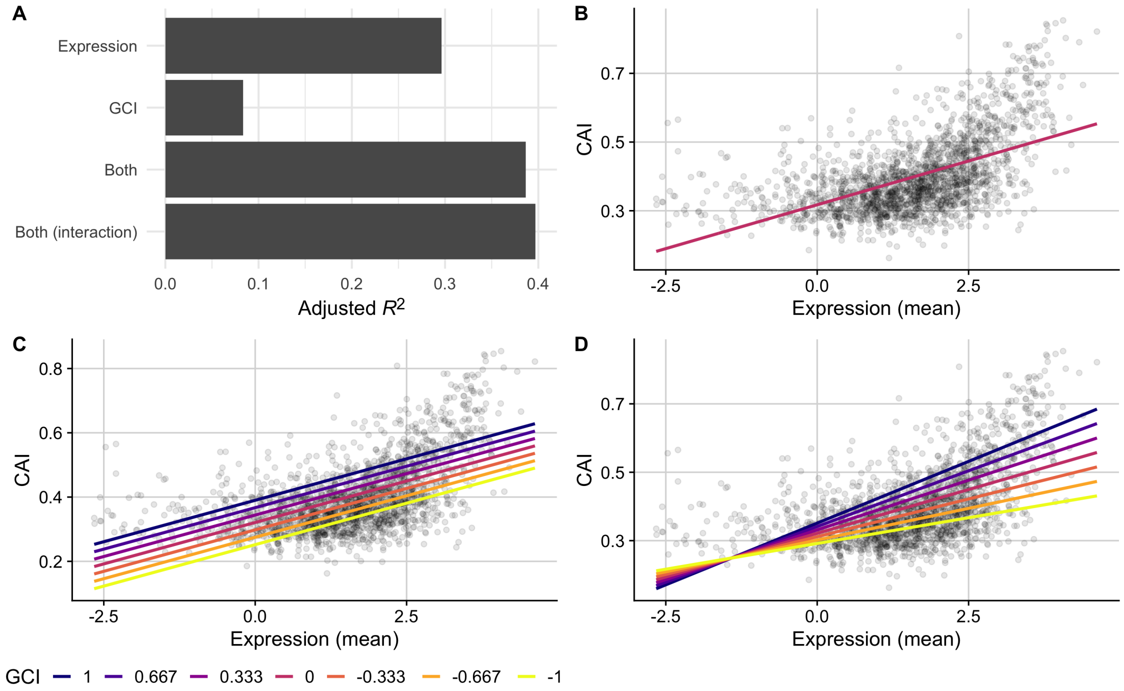

Supplementary Figure S13: **Codon Adaptation Index (CAI) is partially predicted by Growth Correlation Index (GCI) in the *E. coli* protein data set.** A comparison of the predictive ability (measured as R^2 after adjustment) of linear models that use either: 1) mean expression values, 2) GCI values, 3) both expression and GCI values, or 4) both values with an interaction term, to predict CAI. (B, C, D) CAI against mean expression for the top 3 performing models with observed values for each gene shown as points and model predictions as lines. The fit of model 1, which predicts CAI using only mean gene expression values, is shown with one line (B), while models 3 and 4 are shown with several lines colored by potential fixed GCI values (C and D, respectively). VIF (variance inflation factor) between mean expression and GCI is calculated to be 1.00.

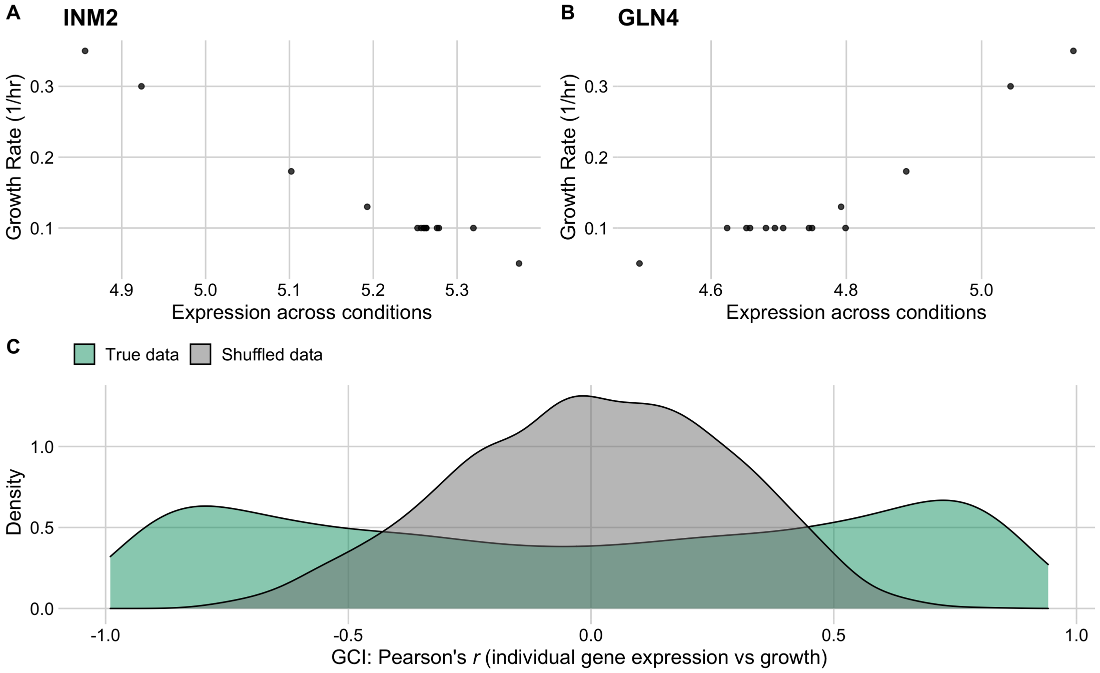

Supplementary Figure S14: **Individual gene expression across conditions variably correlates with growth rate in the *S. cerevisiae* RNA data set.** The Growth Correlation Index (GCI) measures the correlation between growth rate and expression for individual genes. The top row shows the two genes with the most negative (INM2, A) and most positive (GLN4, B) correlation between growth rate and expression across all conditions. (C) The distribution of GCI values for all genes (shown in green) against a reference data set with permuted expression and growth data (grey).

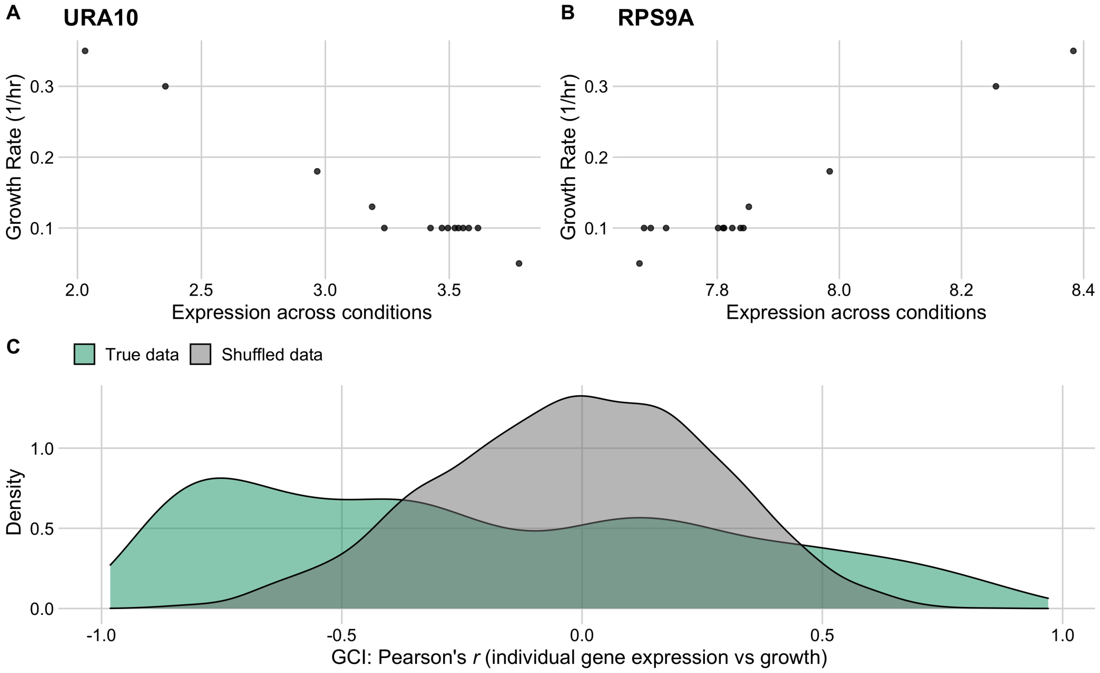

Supplementary Figure S15: **Individual gene expression across conditions variably correlates with growth rate in the *S. cerevisiae* protein data set.** The Growth Correlation Index (GCI) measures the correlation between growth rate and expression for individual genes. The top row shows the two genes with the most negative (URA10, A) and most positive (RPS9A, B) correlation between growth rate and expression across all conditions. (C) The distribution of GCI values for all genes (shown in green) against a reference data set with permutated expression and growth data (grey).

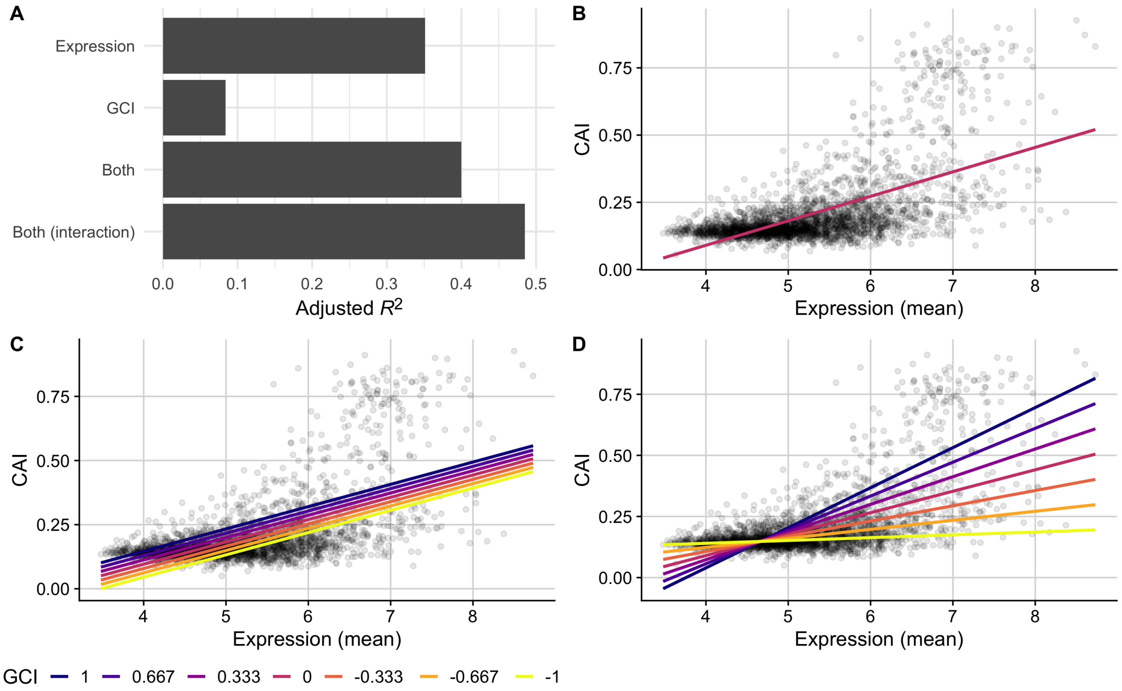

Supplementary Figure S16: **Codon Adaptation Index (CAI) is partially predicted by Growth Correlation Index (GCI) in the *S. cerevisiae* RNA data set.** A comparison of the predictive ability (measured as R^2 after adjustment) of linear models that use either: 1) mean expression values, 2) GCI values, 3) both expression and GCI values, or 4) both values with an interaction term, to predict CAI. (B, C, D) CAI against mean expression for the top 3 performing models with observed values for each gene shown as points and model predictions as lines. The fit of model 1, which predicts CAI using only mean gene expression values, is shown with one line (B), while models 3 and 4 are shown with several lines colored by potential fixed GCI values (C and D, respectively). VIF (variance inflation factor) between mean expression and GCI is calculated to be 1.01.

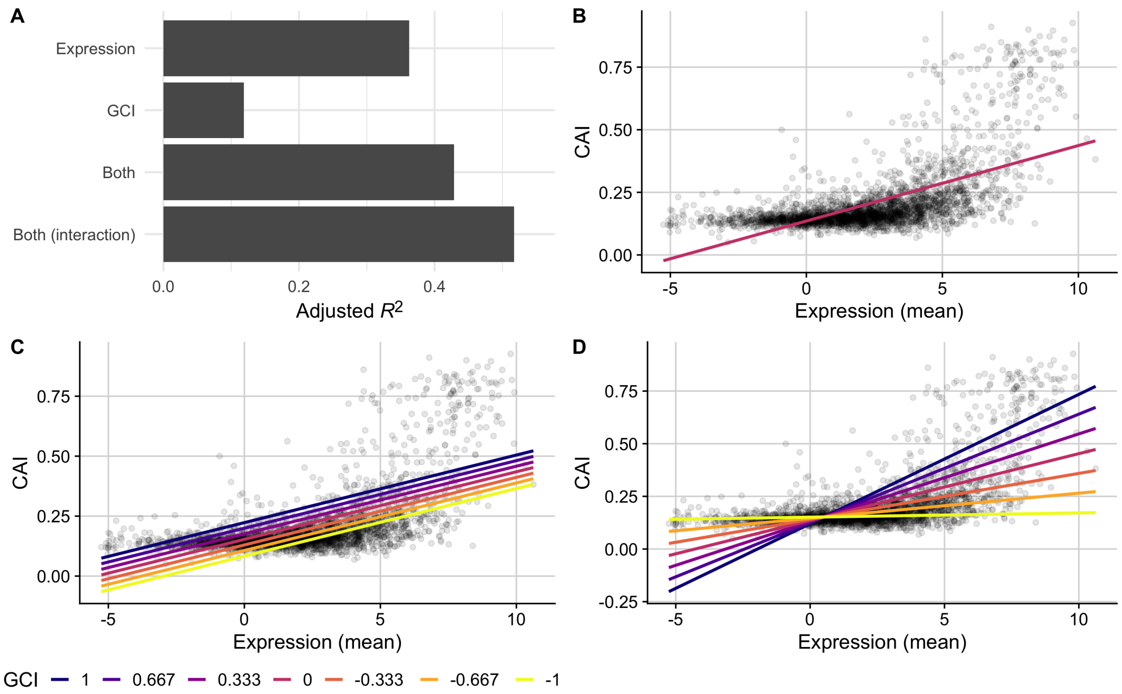

Supplementary Figure S17: **Codon Adaptation Index (CAI) is partially predicted by Growth Correlation Index (GCI) in the *S. cerevisiae* protein data set.** A comparison of the predictive ability (measured as R^2 after adjustment) of linear models that use either: 1) mean expression values, 2) GCI values, 3) both expression and GCI values, or 4) both values with an interaction term, to predict CAI. (B, C, D) CAI against mean expression for the top 3 performing models with observed values for each gene shown as points and model predictions as lines. The fit of model 1, which predicts CAI using only mean gene expression values, is shown with one line (B), while models 3 and 4 are shown with several lines colored by potential fixed GCI values (C and D, respectively). VIF (variance inflation factor) between mean expression and GCI is calculated to be 1.02.
